# Flux and separation of magneto-active superballs in applied fields

**DOI:** 10.1039/d1cp03343c

**Published:** 2021-09-29

**Authors:** Martin Kaiser, Sofia S. Kantorovich

**Affiliations:** University of Vienna, Physics Faculty/Research Platform MMM Mathematics-Magnetism-Materials Vienna Austria martin.kaiser@univie.ac.at; Faculty of Physics, University of Vienna Boltzmanngasse 5 1090 Vienna Austria; Ural Federal University, Russian Federation/MMM Mathematics-Magnetism-Materials Lenin Av. 51, Ekaterinburg 620000 Vienna Austria

## Abstract

The term “active matter” describes a class of out-of-equilibrium systems, whose ability to transform environmental to kinetic energy is sought after in multiple fields of science. A challenge that still remains is to craft nanometer-sized active particles, whose motion can be efficiently directed by externally applied bio-noninvasive stimuli. Adding a magnetic component and therefore being able to direct the motion of active nanoparticles with an applied magnetic field is one of the promising solutions in the field. In this study, we employ molecular dynamics simulations to predict an external field-induced flow that arises in mixtures of magneto-active nanosized cubic and spherical particles with distinct mutual orientations between magnetization and propulsion. We explain why the flux of the suspended particles in the field direction does not only depend on the angle between the active force, driving a particle forward, and the orientation of its magnetization, but also on particle shape and inter-particle interactions. Our results show that by tuning those parameters, one can achieve complete separation of particles according to their magnetization orientation. Based on our findings, along with optimizing the cargo properties of magneto-active nano-units, the actual composition of the magneto-active particle suspension can be characterized.

## Abstract 2

A suspension of active-magnetic cubes with different magnetisation orientation, *d*_o_, separate in an applied magnetic field.

## Graphical abstract

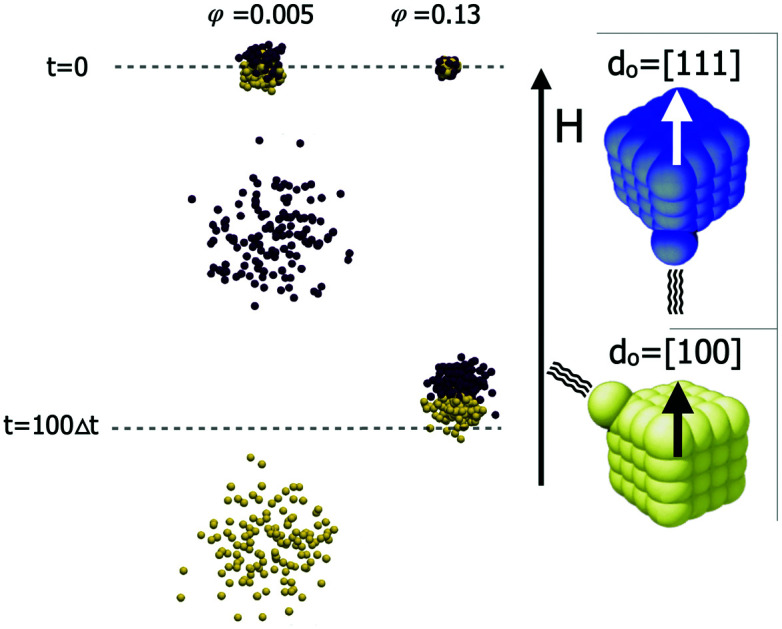

Active matter systems have gained increased interest during the past decade because of their ability to convert environmental energy into persistent motion. Multiple artificially created active particles crafted on the micro- or nanoscale were already designed to utilize a variety of mechanisms to achieve self-propulsion, including concentration gradients (chemotaxis),^1,2^ application of magnetic fields (magnetotaxis)^3–7^ or self-thermophoresis by defocused lasers.^6,8,9^ Nowadays, the active velocity achieved by macron sized particles can reach as high as 10 mm s^−1^ ^10^ and even nanometer sized particles reach velocities in the range of 100 μm s^−1^.^11^

New characterization techniques have revealed various phenomena occurring in active matter systems, ranging from unique swarming and collective behavior^12–15^ that have been intensely investigated experimentally and theoretically, to complex mechanisms such as inverse solidification in crystals^16^ and ratchet effects.^17^ Other fields of increased interest are micro- and nano-scale cargo transport, where self-propelled particles often provide highly suitable candidates for its realization,^18,19^ clogging and unclogging of channels and tubes.^20^ Another possibility that opens up is to use active-particle powered micron sized gears^21^ to create micro-fluidic devices. Though these are just a fraction of applications that researchers envision for active matter systems, they share with a multitude of other applications the following limitation: the processes responsible for their performance can be either enhanced, executed more efficiently or even made at all possible by utilizing particles that do not perform random active motion, but rather directed motion through manual inputs. Ideally, such particles would take an easy to apply input that for biological and medical applications should also be non-invasive to the surrounding tissue, that allows the applicants to fully control the active force of the particle to point in a desired direction.

In this respect, the nanoscale imposes an additional variety of challenges, not least because of the high rotational diffusion that active nano-particles experience due to thermal fluctuations that needs to be tamed in order to achieve directed motion. This is generally an issue when it comes to the above-mentioned applications. Until now, the low effort fabrication of nanometer-sized systems that meet the requirements of being active and controllable remains a task to be solved, as either functionalizing a passive particle to become active, or making an active particle susceptible to external drives is a demanding endeavor. Additionally, systems that do meet the requirements include a complex mix of forces and mechanisms which need to be studied intensely to ensure detailed insight.

Examples of nanoscale systems that can be efficiently controlled by external stimuli and where the forces can be several times higher than the thermal fluctuations include suspensions of magnetic nanoparticles in magnetopassive liquid carriers. Magnetic forces in those systems have been shown to result in reversible formation of clusters, whose topology depend on the shape of the particles and their concentration, and can vary from chains, rings and branched-like aggregates for spheres, to various lattice and carpet-like structures for ellipsoids and cubes in zero-field.^22–26^ One can even further broaden the set of topologies, by altering the internal structure.^27–29^

The shape that embraces the range from spheres to cubes by changing only one parameter is a superball. In the equation1
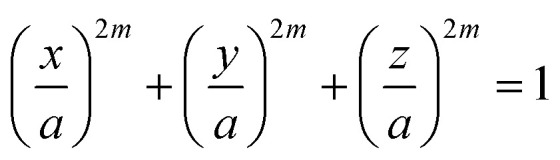
the parameter *m* – so-called cubicity – can vary from unity leading to a sphere of radius *a* to infinity, describing a perfect cube with the side length *a*. In fact, *m* ∼ 2 corresponds to a particle whose cubicity is overwhelming.^26,30^ The anisotropy of cubic particles renders the intrinsic orientation of the magnetization as a crucial factor for the orientation at which a free energy minimum is achieved: cubes in which the dipole points along the [100] crystallographic axis tend to form linear chains, regardless of whether a magnetic field is applied or not; if the magnetization orientation lies along the main diagonal [111], the particles cluster in lattice-like structures in zero-field and form zig-zag structures in field.^31^

In this manuscript, we investigate the suspension of monodomain magnetic superballs with rigidly attached spherical active particle to one of their corners, aiming at finding a system that is easy to direct with a homogeneous magnetic field and describing the mechanisms that are responsible for the latter efficiency. For a system of magnetic particles the control parameters capable of affecting the propulsion are the particle shape that defines both interparticle steric and magnetic interactions, and the particle magnetic properties that determine the coupling of each particle and an applied field, as well as the strength of the magnetic interparticle interaction.

A simulation snapshot of the *m* = 2.3-superball with a motor is shown in Fig. 1(a). This model has previously revealed the dependence of the propulsion direction on the mutual orientation of the magnetisation and active force for an exemplary system. Its nanosized experimental realization is provided in Fig. 1(b).^32^ In the latter study we focused on highly diluted suspensions of single-domain cobalt ferrite (CoFe_2_O_4_) nanocubes with the side of approximately 20 nm decorated with a smaller platinum (Pt) nanoparticle attached to its corner. The value of the particle magnetic moment in the experiment was approximately 1.41 × 10^−18^A m^2^. The probability of the intrinsic magnetization of such a cube to point along the [111] axis is higher than that along [100], but none of them can be excluded. Depending on the latter orientation, under the condition of infinite dilution, a single cube propels either along or opposite to the direction of an applied magnetic field. Although below we stick to this very experimental realization to choose the simulation parameters, as long as the shape of the magnetic colloids is described by eqn (1), the active motor is attached in such a way that the propulsion velocity vector points along the line connecting the surface and the superball center of mass, the colloidal magnetization can be approximated by a point dipole that points at a given angle to the propulsion velocity, and the size of the particle is sufficiently small for thermal fluctuations to matter, our approach is fully applicable and conceptually similar results are expected.

**Fig. 1:**
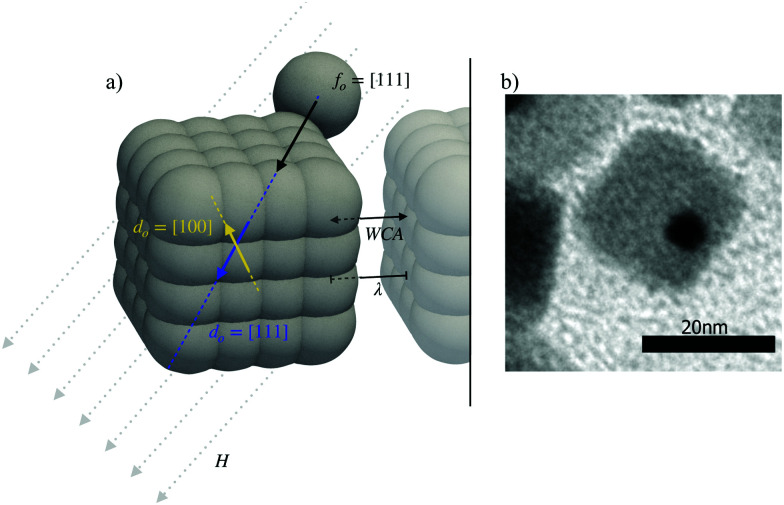
(a) Schematic of a magneto-active superball with *m* = 2.3. The active portion is rigidly attached to the top right corner. The schematic marks the forces acting on this particle in suspensions: the active force *f*_0_ always points towards the center of the cube, where a dipole *d*_0_ sits pointing either along the [100] or [111] axis of the cube. The dipole interacts with the uniformly applied magnetic field *H*, as well as with other dipolar cubes through the inter-particle magnetic interactions *λ*. Between the particles a steric repulsive Weaks–Chandler–Andersen (WCA) potential also acts. (b) Experimental nanosized realization with the platinum colored in black, and the cobalt-ferrite cube colored in gray. The figure has been taken from a private correspondence with the Schmidt Group, University of Cologne.

In a suspension, where magnetic superballs with different magnetisation orientation with respect to the propulsion velocity are present, along with Zeeman coupling, the inter-particle interactions shall affect the diffusivity of individual units. The latter in its turn will depend on the particle cubicity. In order to investigate the macroscopic fluxes arising in variously composed mixtures of magneto-active superballs and the microscopic mechanisms leading to those fluxes, we perform extensive molecular dynamic simulations, whose results are presented below in the following order. First, we discuss the flux in various directions with respect to an applied field and flux dependence on the suspension microscopic composition, showing the separation of the suspended particles at low concentrations according to the orientation of their magnetization. In order to clarify the role of magnetic interactions, we additionally perform the analysis of the systems with the magnetic inter-particle interactions artificially switched off, but the coupling to the field present. Next, we focus on the influence of shape on the separation by comparing the system of *m* = 2.3-superballs to that of a different limiting case of *m* = 1, namely, spheres. After that a brief summary follows. The manuscript ends with the section where we provide methods and parameters used in the simulations, as well as the connection of those parameters to one of the possible experimental realizations.

## 1 Results and discussions

### 1.1 Particle flux in suspensions under an applied field

In order to get insight into the suspension's behavior, we investigated mixtures including [100] and [111] highly cubic *m* = 2.3-superballs that, for brevity, below we address as cubes, in different proportions preserving a total volume fraction *φ*, starting from a monodisperse system of [111] type and gradually replacing a part of the cubes by their [100] counterparts until a full inversion. The value of *φ* is varied between half a percent and 13 percent.

**Fig. 2:**
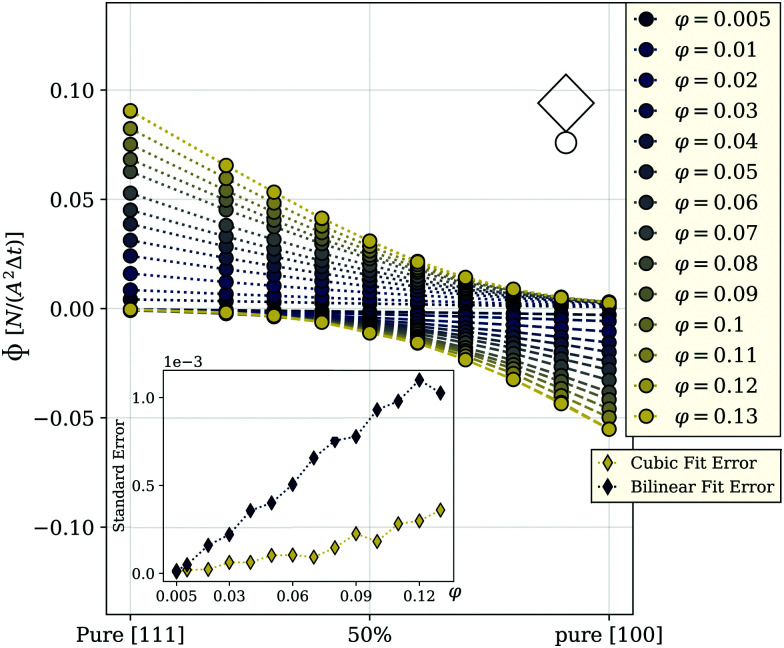
Flux *Φ* as a function of the mixing ratio between [111] and [100] magneto-active cubes (*m* = 2.3-superballs). The 50 percent mark in the center of the axis indicates a 50/50 ratio between the two cube types. The color encodes the volume fractions *φ* of the investigated systems as shown in the legend. The dotted lines connect the points representing the flux in the field direction, and dashed lines connect the points representing the flux in the direction opposite to the field; a little cube in the corner is to distinguish the flux plots provided below in the manuscript. Magnetic interaction between the cubes is ∼3.6 times stronger than the thermal energy. The plot is obtained at room temperature. Inset: Standard error of data points from this figure to a bilinear (blue) or a cubic (yellow) fit function. The error is calculated as the standard deviation of the data points from the best fit for a given volume fraction. The dotted lines are guides for the eye.

The ability to control the propulsion direction by an applied field forms the basis for the transport applications,^33,34^ and the best possible way to quantify the control efficiency is to measure the particle flux in the given direction. The advantage of using active particles in such applications lies in the fact that no gradient field is required to create a flux. Thus, we use a homogeneous applied field *H⃑*, *H⃑* = 9.22, that is for the case of nanosized cubes equivalent to approximately 26 millitesla, and measure the flux, *Φ*, in the direction of the field through a plane with unit area *A*^2^ perpendicular to *H⃑*. The area is determined by the volume fraction considering a fixed number of particles in all simulations. To calculate *Φ*, we count the number of particles that cross the plane during a time interval Δ*t*, where Δ*t* is the time between two simulation frames. The results are shown in Fig. 2.

This figure shows the components of the flux that are in field and opposite to the field direction. The flux in the field direction is largest for a pure [111] system, decaying monotonously with increasing the fraction of [100] particles in the system. The behavior of the flux opposite to the field behaves exactly inverse, growing monotonously at larger mixing ratios. This can be explained by reviewing the behavior of single [111] and [100] cubes in the field: we have shown that [111] cubes at infinite dilution tend to follow the magnetic field direction, while the [100] cubes, under the same conditions, mainly propel into the opposite direction. We can see that the flux of a pure bulk system of [100] cubes in the field direction is nearly zero, meaning that the individual behavior is preserved in an ensemble. For any *φ*, the flux in the field direction reaches its maximum for pure [111] systems. Even though all curves exhibit monotonic decay, the functional behavior of the curves changes from linear at low *φ*, to strongly non-linear for total volume fractions above approximately three percent. In Fig. 2, with symbols we plot the simulation data, while the lines are cubic polynomial fits. Up to *φ* = 0.02, the value of the fitting coefficient in front of the third power is very small, however, when the concentration grows further, this coefficient increases by two orders of magnitude. Up to *φ* = 0.02, the value of the fitting coefficient in front of the third power is very small, however, when the concentration grows further, this coefficient increases by two orders of magnitude. In order to fully exclude the possibility of a bilinear behavior of the flux at low and high densities, we also tried to fit the data with a bilinear function, having the same number of free parameters as the cubic polynomial fits. However, the mean deviation of data points to the best bilinear fit is significantly higher than that to a cubic function which is shown in the inset of Fig. 2. It is therefore worth understanding in detail the mechanisms that are responsible for this *φ*-dependent behavior of *Φ*, because its unique correspondence to the composition mixture makes the flux a promising tool for gathering information about the granulometry of magneto-active cube suspensions in experiments, where the fractions of particles with different intrinsic orientation of magnetization are not *a priori* known. The system under study has three main ingredients: magnetic and steric inter-unit interactions and unit activity. In a steady state, the activity in combination with an applied magnetic field, lead to the flux shown in Fig. 2, but on its own does not affect the coupling between the particles (units), or of particles and the field. Below, we split the contributions of the remaining two ingredients.

### 1.2 Influence of magnetic interactions on the particle flux

**Fig. 3:**
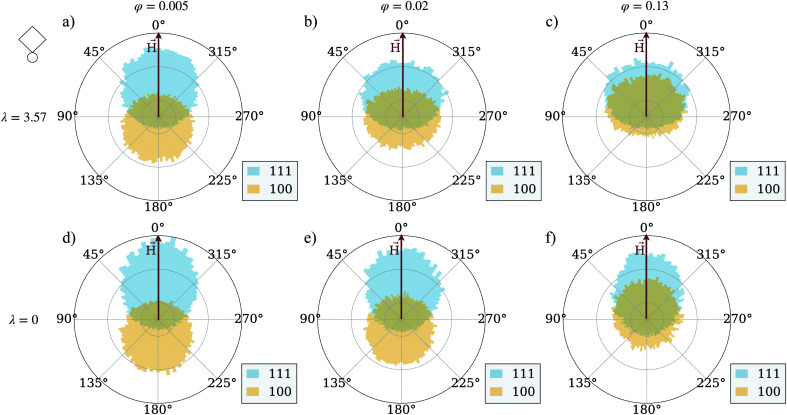
Polar distributions of the track-angle (*H⃑*, in red, aligned with the 0 line) – for the [111]-particles (in light-blue) and [100]-particles (in light-yellow). In (a)–(c) the magnetic interaction is ∼3.6 times stronger than the thermal energy; in (d)–(f) the magnetic interactions are set to zero. The columns determine the total volume fraction of the system, ranging from 0.005 in (a) to 0.02 in (b) to 0.13 in (c). For all plots, the interaction with the field is ∼9 times stronger than the thermal energy; the mixing-ratio is 50 percent.

To show the influence of magnetic interactions of the units on their trajectories, we calculate the angle enclosed between a vector pointing from one trajectory point to the next and the magnetic field vector, the track-angle. This quantity is averaged over all [100] and [111] particles respectively at a fixed mixing-ratio of 50 percent. In this way, we can quantify the trajectory of the particles in a comprehensive way and look at trends for the preferred motion direction of both unit types. Fig. 3 shows a series of track-angle distributions for suspensions of magnetically interacting particles, and those where the magnetic inter-particle interaction is manually put to zero, preserving however the coupling of each particle to the applied field. The latter system is usually addressed as an ideal Langevin superparamagnetic gas. The qualitative behavior follows the same scheme in both cases independently from *φ*, but quantitative effects are evident. At low particle concentrations (compare Fig. 3(a) and (d)), interacting magnetically or not, the [111] particles travel in the direction of the magnetic field, while [100] particles travel in their preferred direction opposite to the field. The reason is that the units are hardly interacting at such low concentrations. However, at higher concentrations the [100] particles partially switch the direction of their motion and start moving along the magnetic field, but at a lower rate than the [111] particles. This effect is more pronounced for the interacting systems shown in Fig. 3(b) and (c) than for their respective non-interacting counterparts in Fig. 3(e) and (f). This suggests that at higher concentrations [111] particles magnetically exert a drag on the [100] particles which is strong enough to turn around the particles motion. The magnetic interactions hence are at least one of the key factors when it comes to flux increase at higher concentrations and a non-linearity of curves in Fig. 2. Below we check the impact of unit shape.

### 1.3 Influence of particle shape on the particle flux

The influence of particle shape is twofold in our systems. Firstly, the purely mechanical steric interactions arising from the cubic shape and second, the shape induced difference in magnetic interactions between the units.^35,36^ Both have to be taken into account separately. To do so, we replace the initially cubic magnetic part of the swimmer unit with a magnetic sphere, *i.e.* a superball with *m* = 1, the magnetization orientation of which is either coaligned with the eigen propulsion velocity as in the case of [111] cubes, or is perpendicular to the latter, similar to cubes with the magnetic moment along [001] (for details, see the Introduction section, Fig. 1). Importantly, simulating a very cubic particle and a sphere allows us to span a broad range of convex shapes and draw broader conclusions.

**Fig. 4:**
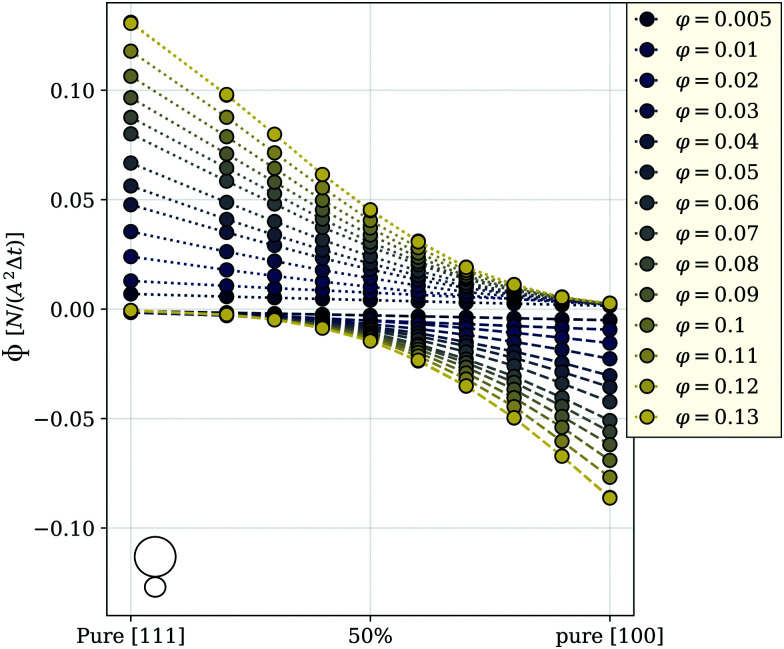
Flux *Φ* as a function of the mixing ratio between [111] and [100] magneto-active spheres. The 50 percent mark in the center of the axis indicates a 50/50 ratio between the two cube types. The color encodes the volume fractions *φ* of the investigated systems as shown in the legend. The dotted lines connect the points representing the flux in the field direction, dashed lines connect points representing the flux in the direction opposite to the field; a little sphere in the corner is to distinguish the flux plots provided above in the manuscript. Magnetic interaction between the spheres is ∼3.6 times stronger than the thermal energy. The plot is obtained at room temperature.

**Fig. 5:**
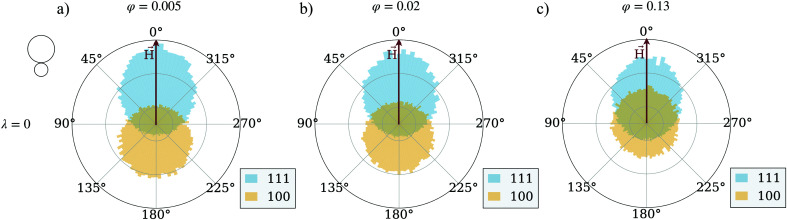
Polar distributions of the track-angle (*H⃑*, in red, aligned with the 0 line) for the [111]-particles (in light-blue) and [100]-particles (in light-yellow). The magnetic interactions are set to zero. The volume fraction of the system ranges from 0.005 in (a) to 0.02 in (b) to 0.13 in (c). For all plots, the interaction with the field is ∼9 times stronger than the thermal energy; the mixing-ratio is 50 percent.

Fig. 4 shows the flux for those spherical particles under the same conditions as in the cubic case presented above in Fig. 2. First of all, the directionality of the in-field motion based on the mutual orientation of the velocity and magnetization holds also for spherical units, as well as the overall *φ*-dependent behavior that remains very similar to that of the cubes, thus for any superball. The flux in the suspensions of spheres is, however, higher at all volume fractions compared to their cubic pendant, directly indicating the influence of particle shape. To elaborate on the impact of the steric interactions, we compare Fig. 3(d)–(f) for magnetically non-interacting cubic units to its equivalent for spheres. The corresponding polar plots are shown in Fig. 5.

**Fig. 6:**
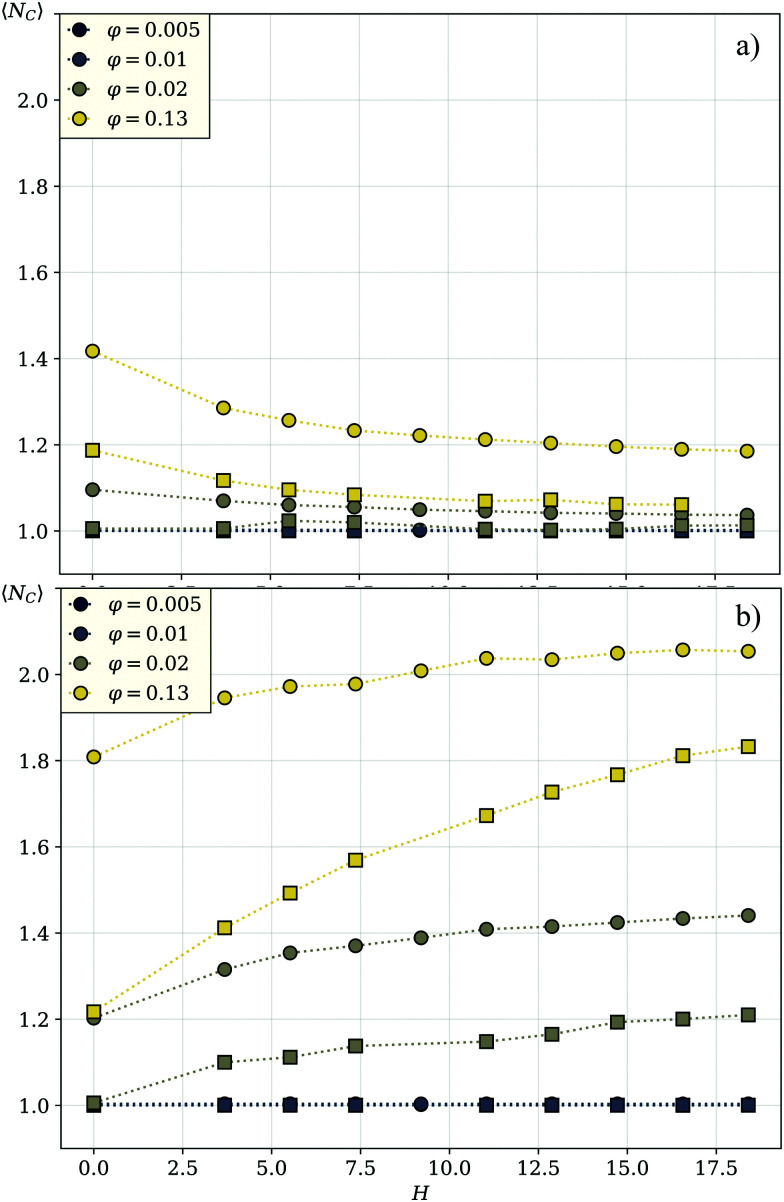
Average cluster sizes in a pure [111] system in (a), and a pure [100] system in (b). The color encodes the volume fraction, the symbol shape encodes the shape of the particles in the system – circles for spherical particles, squares for cubic ones. Note that the line for volume fraction *φ* = 0.005 is not visible as it overlaps with the curves for *φ* = 0.01.

The difference between Fig. 3 and 5 is marginally small and only at the highest investigated particle density, a slightly stronger drift towards the magnetic field direction can be detected in the cube systems. In general, as estimated from the simulations of 50 : 50 mixtures, the probability of a [001] cube to be moving along the field is eight percent higher than that for a [001] sphere. This shows that the steric interactions alone do not give us a sufficient explanation for the higher flux in spherical systems. We are therefore left to explore the magnitude of clustering in both systems, as we have established before that the magnetic interactions are crucial for the flux behavior of the cubic units. Fig. 6 compares the average cluster sizes for pure systems of cubes (plotted with squares) and spheres (plotted with circles) with magnetization orientation [111] in (a) and [100] in (b). Here, we can see how the shape of the particles comes into play. For both cases, [100] and [111], spheres cluster significantly more than cubes over the range of applied field strengths and volume fractions. The exceptions to this are the two lowest investigated fractions *φ* = 0.005 and *φ* = 0.01: for those we see little to no clustering independently from the unit shape, 〈*N*_C_〉 ∼ 1. For both spheres and cubes, this results in the patterns shown in Fig. 3 and 5. At low densities, particles are not affected by magnetic correlations and can move rather freely in their preferred directions. As soon as the systems get dense enough for inter-particle magnetic correlations to start playing a role (*φ* ∼ 0.02) or even more dense for the self-assembly to occur, the directed motion of both the [100] and [111] units is impeded. In other words, in mixtures of active units with different intrinsic magnetization orientation, the directionality of component motion is maximized for dilute systems.

### 1.4 Separation of the [111] and [100] type superballs

**Fig. 7:**
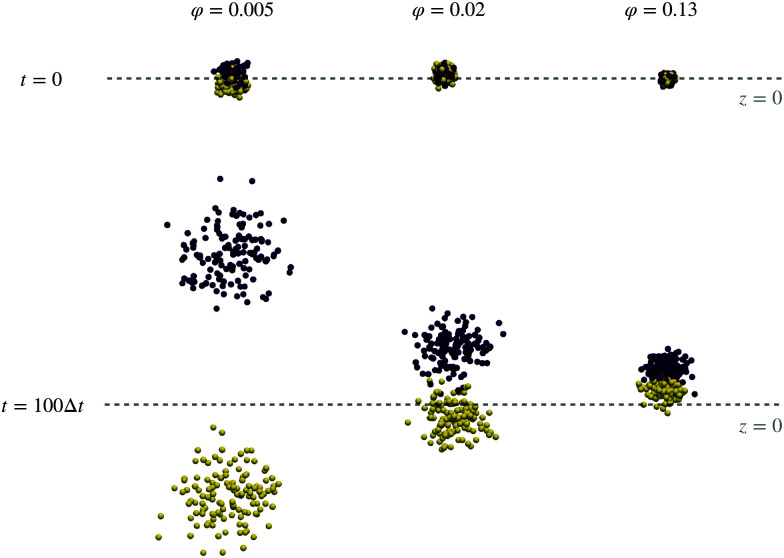
System snapshots of a dipolar-active cube system at 50 percent mixing ratio. Each dot represents the center of mass of a cube. [111] particles are colored in dark-blue, [100] particles in yellow. The top row shows initial configurations at three volume fractions (increasing from left to right) at simulation time *t* = 0. The bottom row shows the same systems at a later time *t* = 100Δ*t*. The dashed line marks the plane of the simulation box where the *z*-coordinate is 0.

Imagine an experiment, in which a drop of an active magnetic superball suspension is put in the middle of a vessel, filled with carrier liquid, and a magnetic field is applied. From the findings above, if the concentration of the magnetic material is low enough for magnetic correlations to not hinder the transport in the preferred directions, we will see a separation of the two particle types according to the mutual orientation of the magnetisation and propulsion velocity. This effect is visualized in Fig. 7, in the left most column. Here, at time zero (top) two types of superballs are fully mixed and after 100 simulation steps the system is fully separated. A different dynamics is observed in the middle row, in which the system with border-line magnetic particle concentration is presented: After 100 time steps only partial separation of superball types in space is observed. Even slower separation is observed on the right of Fig. 7. This, however, does not mean that the full separation will not occur at any concentration after a sufficient amount of time. In fact, at some moment due to partial slow separation, the local concentration will decrease enough to speed up the dynamics of both species. It is worth noting that to accurately understand Fig. 7 it is important to know that the unfolded positions from PBC simulations are plotted (see the Methods section), resembling infinite bulk systems. Therefore, in simulations, systems at high concentrations would continuously migrate in the same direction without the loss of the effect due to full separation of the two particle types.

## 2 Conclusion

By performing Langevin Dynamics simulations, we investigated the behavior of various bulk mixtures of superball magnetoactive nanoparticles, whose intrinsic magnetization can be either co-aligned with the propulsion velocity and point along the cube main diagonal (addressed as [111] superballs), or point perpendicular to the velocity [100]. Under an applied magnetic field, the flux of the suspension in the field direction is determined by the mixing ratio of the two latter particle types. The investigation of flux is of high relevance, as it is the ability to direct the nanoscale active objects with external stimuli that defines their capacity for serving as cargo in micro- and nano-fluidics. The flux at higher concentrations of [111] superballs increases as the dipole tends to align the propulsion direction with the magnetic field, while the [100] particles reorient the propulsion direction to point in the opposite direction of the field. Internally, the flux is influenced by the inter-particle magnetic interactions that depend on the orientation of the dipole inside the particles, as well as their shape. We find that at higher concentrations, particles moving opposite to the field experience a high enough drag from the particles moving along the field so that the whole suspension uniformly moves in the in field direction. The bigger part of this effect is attributed to the magnetic interactions between the particles, with a smaller contribution from the mechanical steric interactions. Both of the latter are verified by first manually switching off the magnetic inter-particle interactions, and secondly by spanning the range of cubicity from highly cubic to perfectly spherical superballs, leaving other parameters unchanged. The stronger clustering as well as shape contributions lead to an overall higher flux for spheres than for cubic particles. Finally, the presented results show us that mixtures of [111] and [100] type superballs separate. Moreover, in dilute enough systems, where the inter-particle magnetic interactions are not dominating the mobility, this happens faster than in concentrated ones. A broader conclusion can be further drawn by noting the underlying class of systems of our models. The particle models we used in this study are extreme cases of superball particles, where one case is highly cubic and the other perfectly spherical. We therefore expect the qualitative behavior of the results shown to hold true for any particle satisfying the superball equation. Results for low particle concentrations, we even expect it to hold true for any particle shape, as long the the active force and dipole are at an appropriate angle since the interparticle interactions, whether steric or magnetic, are low enough to not interfere with the diffusive processes of the single particles. If the system would be scaled up to bigger particle shapes, the interparticle magnetic interactions and the Zeemann energy would get larger. Based on our results, a higher Zeeman energy means steadier redirection of particles, enhancing the effect of directed motion. Stronger inter particle interactions are likely to even further facilitate the control of the particle flux. Special care has to be taken when it comes to active forces in larger size scale systems. While it is easier to achieve higher active forces and therefore larger Peclet-numbers, hydrodynamic interactions between the particles are known to influence the motion in densely packed and high Peclet-number systems and have to be taken into account appropriately. Based on the above results, measuring the flux provides a tool for the experimental characterization of magneto-active nanoscale systems, for which the internal composition of the particles is not *a priori* known.

## 3 Active magnetic nanocubes – methods

### 3.1 Numerical model

We performed three-dimensional molecular dynamics simulations in the canonical ensemble to investigate the behavior of suspensions of magnetic-active nanocubes in an applied magnetic field. All simulations were executed using the simulation package ESPResSo.^37^ The underlying equation that is numerically integrated to get the discrete trajectories of the particles is the Langevin equation of motion, that for translational degrees of freedom reads2
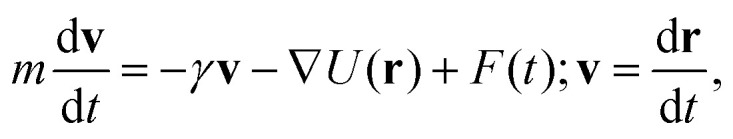
including the quantities: particle mass *m*, particle position *r*, particle velocity vector *v*, friction coefficient *γ*, the gradient of any interaction potential acting on the particle ∇*U*(*r*) and a random force *F*(*t*). The latter should be Gaussian distributed according to Ornstein and Uhlenbeck,^38^ having independent components with magnitude *D*_p_, and *δ*-correlated time dependence,3
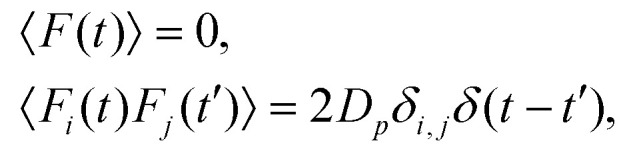
where *i* and *j* can take the indices *x*, *y* and *z* of the spatial directions. This random force represents implicitly the effects of the thermal fluctuations of the background fluid at a temperature *T*, providing the possibility to efficiently produce simulation data for a system at constant thermal energy *kT*, where *k* is the Boltzmann constant. Expressions analogous to (2) and (3) are also integrated for the rotational degrees of freedom. All integrations are performed in cubic simulation boxes with periodic boundary conditions applied in order to mimic the bulk.

The approach used to represent the anisometry of our system is the so-called “raspberry model”.^39,40,41^ This approach is based on the usage of a rigid arrangement of spherical building blocks to effectively reproduce any body shape. The spherical building blocks (including the active particle sitting at the corner) are connected to the center of mass of the cube, so that all forces and torques are transferred to the latter. Here, we build on the model used by Donaldson *et al.* for simple magnetic nanocubes.^35,36,42^ The size, shape and mass of the raspberry grains are chosen in such a way that the resulting cube characteristics closely resemble those in the experiment. The number of grains in the raspberry does not directly affect the diffusion of the particles, only its geometry. Fig. 1(a) shows the raspberry structure of the cubic active unit used in our simulations. The point dipole is placed in the center of the cube. Its relative orientation *d*_o_, defined with respect to the corner at which the active particle is fixed, is either [111] (along one main diagonal of the cube, pointing opposite to the reference corner) or [100] (pointing to the center of one of the faces of the cube that includes the reference corner). The self-propulsion force of the active sphere sitting at the reference corner is constant and pointing towards the center of the cube, thus, parallel to the dipole moment for the case [111] and perpendicular to it for [100]. In simulations, the value of the thermal energy *kT* is chosen as 1. The diameter of a single raspberry grain is *σ*_R_ = 1, resulting in a total height of the cube *h* = 2.5 and a cubic shape-parameter *m* = 2.3. The resulting volume *V*_c_ of a single cube-unit is therefore the volume of the cube itself, plus the volume of the active particle sitting at the corner: *V*_c_ = 13.7. This also determines the box-size *V*_B_, given a volume-fraction *φ* and a fixed number of particles in every simulation *N* = 256, *via* the formula *φ* = *NV*_c_/*V*_B_. The mass of the cube has been chosen by assigning a virtual mass of 1 to each raspberry grain, which results in a total mass of *m*_c_ = 58 for each cube unit (58 grains per unit).

The interaction of the cube magnetic moment, represented as a point dipole *d⃑* with a fixed magnitude, with the applied magnetic field, *H⃑*, is given by the classical Zeeman potential

4 *U*_*z*_ = −*d⃑*·*H⃑*,

In all cases, the field is applied along the *z*-axis of the laboratory reference frame (related to the orientation of the simulation box).

The dipoles interact *via* the dipole–dipole interaction potential,5
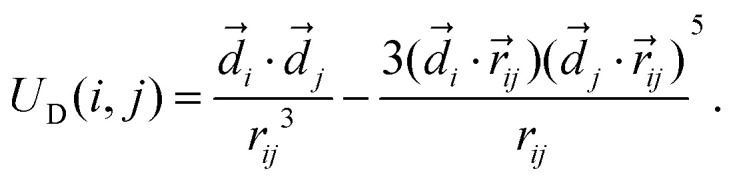
This expression describes the magnetic interactions between two point dipoles *d⃑*_*i*_ and *d⃑*_*j*_. For optimization of the long-range forces calculations, we use the dipolar-P3M solver built in ESPResSo. The dipole moments are chosen such that the magnetic coupling parameter *λ* = *μ*_0_|*d⃑*|^2^/4π*σ*_c_^3^*kT* for the ground-state of [111] and [100], respectively, is *λ* = 3.57. Here *k* is the Boltzmann constant and *σ*_c_ is the side length of the cube, which is the distance of dipoles in the ground state configuration for both cases. It is important to notice that the different ground state configurations require a different magnitude of *d⃑* to achieve the desired *λ*. Due to this technical limitation, the minimal Zeeman-energy for a given field is 17 percent smaller for the [111] particles.

Exploiting the Langevin-equation, self-propelled motion in simulations can be represented by a constant force applied along the propulsion axis of the active particle. Through a velocity dependent friction, an active particle attains a terminal velocity *v*_a_. The value of the latter is defined by the balance of this friction and the driving force. This approach is called the Active-Brownian-Particle (ABP) model and is used to study a variety of active matter phenomena in simulations.^43,44^ The Péclet-number which is defined as Pe = *v*_a_/*σ*_c_*D*_r_, *D*_r_ being the cubes rotational diffusion coefficient, gives the ratio of advective to thermal forces. From the experiment, it is possible to estimate a Peclet number for the system, Pe∼ 0.072. As long as our simulations operate dimensional units, the stokesian translational friction coefficient *γ*_t_ can be put to unity under the condition that the ratio between the translational and rotational diffusion coefficients is correct. The latter for a cube is not trivial and we used the friction tensor obtained by Kazuya Okada and Akira Satoh in ref. 45. This resulted in the rotational friction coefficient value of *γ*_r_ = 1.733. For this parameter the terminal velocity of a cube was obtained to be *v*_a_ = 0.1 in simulation units. The time-scale in a simulation can be found by analyzing the diffusion coefficient of a single, non-active cube unit at the otherwise same parameters. For our units, this results in the 400 frames used for obtaining the data of the present article corresponding to 
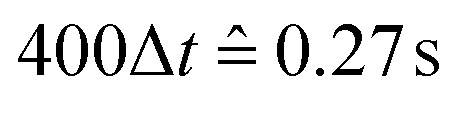
.

In all simulations the integration time step has been chosen to be *τ* = 0.01, ensuring the stability of the integration scheme. The simulation protocol is as follows. First, randomly placed particles are integrated with a steepest-descent scheme until any occurring particle overlaps are eliminated. After that, inter-particle magnetic interactions are switched on and equilibrated for a period of 10^5^*τ*. The magnetic field is then switched on and the particles are given another 10^3^*τ* to relax. This equilibration procedure ensures that from then on the collected data is uncorrelated to the initial position of the randomly placed particles. Measurements on the system happen every 10^4^*τ*, denoted as Δ*t* or just called “frame”, until averages over 3 independent simulation runs can be calculated with at least 400 frames each.

### 3.2 Connection to the experiments

The parameters used in the model represent CoFe_2_O_4_ cubes with a 22 nm magnetic core side length and an additional 2 nm acrylic acid coating. The magnetic cubes with a magnetic moment of 1.41 × 10^−18^ A m^2^ are attached to Pt spheres of 4 nm of diameter. The highest strength of the magnetic field (*H* = 18.5) corresponds to a field of approximately 53 mT. Mechanical properties such as masses and rotational inertia have been taken into account appropriately. The Peclet number of a single unit Pe = 0.072 corresponds to a propulsion velocity of approximately 65 μm s^−1^. All systems are simulated at temperature *T* = 293 K. The magnetic field strength for flux measurements is chosen to realize 85% of the saturation magnetization of the fluid in order to keep the parameters achievable in experiments. Regarding the experimental realization of a flux measurement, single particle tracking measurements would provide a suitable candidate. A possible way would be dark-field microscopy^46,47^ which is capable of detecting single particles of size 10 nanometer or bigger in suspensions using refracted lasers and high-sensitivity CMOS-sensors. The motion of magnetic particles can also be detected with measuring coils through the fluctuating electromagnetic field created by the particles.^48^

## Conflicts of interest

There are no conflicts to declare.

## Supplementary Material
